# Effect of Hypertransfusion on Extramedullary Hematopoietic Compression Mass in Thalassemia Major: A Case Report

**DOI:** 10.5812/iranjradiol.8064

**Published:** 2012-09-17

**Authors:** Mohammadreza Emamhadi, Ahmad Alizadeh

**Affiliations:** 1 Department of Neurosurgery, Guilan University of Medical Sciences, Rasht, Iran; 2 Department of Radiology, Guilan University of Medical Sciences, Rasht, Iran

**Keywords:** Beta-Thalassemia, Hypertransfusion, Spinal Cord Compression

## Abstract

Hereby we report a patient with thalassemia major having extradural cord compression at T3-T9 levels due to a mass of extramedullary hematopoiesis (EMH) tissue, whose treatment was successful with hypertransfusion therapy alone. The patient was a 23-year-old man who had not received regular blood transfusion since two years before admission. He suffered from paraparesis with a history of progressive lower limb weakness for 2 months. MRI of the spinal cord demonstrated thoracic extramedullary hematopoietic mass causing spinal cord compression. The patient demonstrated a significant response to hypertransfusion and improvement in the neurologic status started a few days after treatment. Almost complete resolution of the mass was seen in spinal MRI one week after hypertransfusion. Hypertransfusion seems to be a useful method for treatment of spinal cord compression due to a hematopoietic mass. It may be used as the first line therapy.

## 1. Introduction

Extramedullary hematopoiesis (EMH) is considered as a compensatory reaction in patients with chronic anemia such as thalassemia. Insufficient production of blood elements in the marrow of long bones, ribs and the vertebrae for blood circulation demand is remarkable among these patients which leads to formation and maturation of blood elements in various extramedullary sites such as the liver, spleen and the paraspinal regions of the thorax (1, 2, 3). Among all etiologies of EMH, thalassemia is the most common cause, but intraspinal location is extremely rare, observed in 11% (4) to 15% (5) of the patients. Management of these patients still remains controversial mainly consisting of blood transfusions, decompressive surgery and radiotherapy. This article presents one case of spinal cord compression caused by an extramedullary hematopoiesis mass in a patient with thalassemia major undergoing successful treatment with blood hypertransfusion.

## 2. Case Presentation

**Figure 1:**
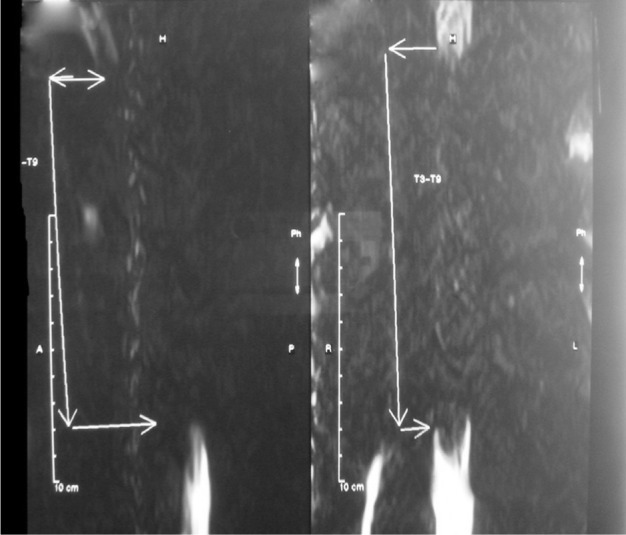
Before transfusion;thoracic myelogram showed an extradural mass lesion that it caused to spinal cord compression atT3-T9 level.

A 23-year-old man with thalassemia major was admitted with paraparesis and a 2-month history of progressive lower limb weakness. The patient had undergone splenectomy 2 years ago and he had not received regular blood transfusion after that. His hemoglobin level was 9g/dl on admission. Neurological examination revealed severe spastic paraparesis (grade 2), hyperactive deep tendon reflexes with sustained clonus in the ankles and a decreased sensation to all modalities from toes up to T4 dermatoma. Spinal cord compression was demonstrated in neurological examination, it was then confirmed by magnetic resonance imaging (MRI). Soft tissue mass on both sides of the thoracic spinal area at T3 toT9 level was revealed in MRI of the thoracic spine without gadolinium injection (sagittal T1, T2 and axial T2) with a configuration of extradural posterior compression (Figure 1).

**Figure 2:**
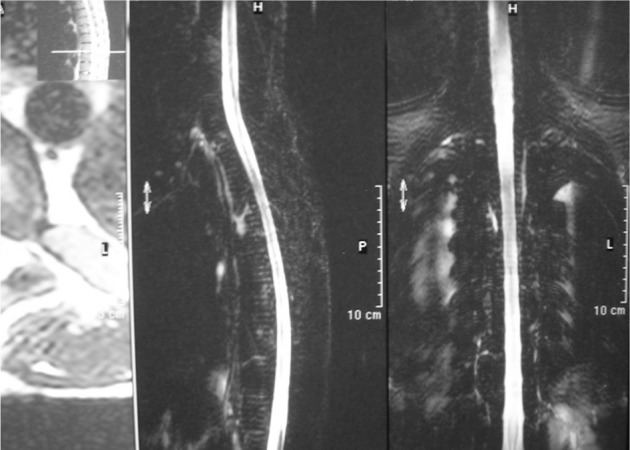
After transfusion, MRI showed complete resolution of the extramedullary mass.

Hypertransfusion was applied for the patient. The improvement was rapid, starting a few days after transfusion. A new MRI of the spine one week after hypertransfusion showed complete resolution of the extramedullary hematopoietic mass (Figure 2). His neurologic examination quickly improved (grade 4) and he was discharged in a good condition two weeks after hypertransfusion.

## 3. Discussion

Thalassemia is a prevalent genetically determined disorder in Iran. EMH is a compensatory phenomenon in these patients that is commonly seen in the abdomen, chest or epidural space (2). In rare cases, the mass effect of EMH within the spinal canal can cause symptomatic spinal cord compression. The pathogenesis of this disease still remains controversial. It may be attributed to the extension of hyperplastic marrow through the thinned out trabeculae related to the proximal end of the ribs. It is also claimed that the remained embryonic cells within the epidural space are transformed into hematopoietic tissue (6, 7). Neurologic signs and symptoms are led by epidural masses via direct compression of the nerve roots and the spinal cord. In the primary clinical course, symptoms may include focal back pain and paresthesia, progressing to sensory impairment, spastic weakness, bladder and bowel dysfunction and abnormal reflexes. Paraplegia or quadriplegia may ensue epideral masses usually resulting in compression of the nerve roots and sometimes the spinal cord. Symptoms include low back pain and paresthesia, while advancing there may be sensory impairment and even paraplegia or quadriplegia and dysfunction of the bladder and bowel (8). According to reports in the literature, symptomatic cord compression most frequently involves the thoracic cord similar to our case; however, the reason of this predisposition is left uncertain (9, 10, 11, 12). The first description of spinal cord compression by EMH dates back to 1954 (Gatto). Since then, about 60 cases have been reported, mainly with intermediate β-thalassemia. Alberti et al. (2001) described two cases with thalassemia and paraparesia resulting from intrathoracic EMH, successfully treated by blood hypertransfusion. They reported their patients did not need any further therapy. Moreover, no recurrences were observed after their treatment during the follow-up period (1). Our patient completely recovered by hypertransfusion therapy alone. Almost complete resolution of extramedullary hematopoietic mass was displayed in the spinal MRI one week after hypertransfusion and the neurological examination was normal. Besides, we did not observe any recurrence.

MRI is the first selected imaging modality for the primary diagnosis of EMH (6, 13). MRI clearly showed the site and extent of the lesion in relation to the spinal canal, which displaced the spinal cord anteriorly. Due to the reason that sagittal sections indicate the exact length of the lesion in the spinal canal, it is considered more beneficial than CT. In T1-weighted MRI, a mass is seen which has a slightly higher signal intensity than that of the vertebral bodies, gadolinium will also help when there is no abnormal mass in the spinal canal on regular imaging (14, 15, 16).

Treatment of cord compression due to EMH is a matter of debate. In emergency cases, intravenous steroids may be used as the temporizing measure until definitive treatment is applied (8). Other therapeutic methods of spinal cord compression due to extramedullary hematopoiesis are as follows: surgical decompression, radiation therapy or transfusion. Although providing tissue, allowing a definitive histological diagnosis along with immediate decompression preventing permanent damage are all advantages of surgery method (6, 11, 17, 18), the risk of bleeding due to the high vascularity of the mass, possibility of incomplete resection and high recurrence rate still exists. Furthermore, many of the anemic patients are not considered as appropriate surgical candidates due to their cardiovascular complications. Radiation is another applicable therapy. This method avoids surgical procedure and associated risks (cardiovascular debility due to anemia and iron overload) (19, 20, 21). However, suppression of bone marrow due to radiation may be observed in already anemic patients. In some patients whose surgery and radiation therapies may cause special risks (e.g. in pregnancy), transfusion has been proven to be successful within which relieving the anemic stress leads to suppression of the hematopoietic tissue. This will especially be effective in patients presenting severe EMH (10).

Cord compression resulting from EMH is a rare complication in patients with thalassemia. An immediate therapy is necessary in the management of patients with cord compression and can result in promising response in different treatment modalities such as surgical decompression, radiation therapy and repeated blood transfusion. However, the appropriate treatment of these patients depends on their status; hypertransfusion seems to be useful which can be regarded as a first-line treatment.
